# Evaluation of glycoprotein Ov8 as a potential antigen for an OvHV-2-specific diagnostic assay

**DOI:** 10.1371/journal.pone.0200130

**Published:** 2018-07-02

**Authors:** Salim M. Alhajri, Cristina W. Cunha, Donald P. Knowles, Hong Li, Naomi S. Taus

**Affiliations:** 1 Department of Veterinary Microbiology and Pathology, College of Veterinary Medicine, Washington State University, Pullman, Washington, United States of America; 2 Animal Disease Research Unit, Agricultural Research Service, United States Department of Agriculture, Pullman, Washington, United States of America; 3 Paul G. Allen School for Global Animal Health, Washington State University, Pullman, Washington, United States of America; National Institute of Animal Biotechnology, INDIA

## Abstract

Gammaherpesviruses in the genus *Macavirus* establish clinically unapparent persistent infections in reservoir species. Transmission of some of these viruses, including alcelaphine herpesvirus 1 (AlHV-1) and ovine herpesvirus 2 (OvHV-2), to clinically susceptible species in the order Artiodactyla can result in malignant catarrhal fever (MCF), a usually fatal lymphoproliferative disease. Serology can be used to identify MCF virus (MCFV)-infected carrier animals. However, all current serological assays utilize AlHV-1 antigens, thus none is specific for OvHV-2. In situations where sheep and other MCFV carriers are present, such as in zoos and game farms, an OvHV-2-specific assay would determine if OvHV-2 is present in the population. In this study, a recombinant protein containing a truncated OvHV-2 Ov8 glycoprotein was expressed and evaluated as a suitable target antigen to specifically detect OvHV-2 infection using an enzyme linked immunosorbent assay (ELISA). A competitive inhibition (CI)-ELISA that detects an epitope conserved among all MCFVs was used to categorize, as positive or negative, sera from 205 domestic sheep. The Ov8 assay showed 100% diagnostic sensitivity, 98.97% diagnostic specificity, 99.07% positive predictive value, and 100% negative predictive value and very high agreement (kappa = 0.990 and 95% CI = 0.971–1.000) with the CI-ELISA. Sera from animals infected with MCFVs other than OvHV-2 did not cross-react with Ov8 (100% negative predictive value). These data support the use of the Ov8 ELISA as an OvHV-2-specific diagnostic assay.

## Introduction

Ovine herpesvirus 2 (OvHV-2) is a gammaherpesvirus in the genus *Macavirus*. It is carried asymptomatically and spread by domestic sheep, which are the well adapted reservoir species. Infection of clinically susceptible animals with OvHV-2 can result in malignant catarrhal fever (MCF), a usually fatal disease of many wild and farmed species in the order Artiodactyla, including bison, cattle and deer [1–3]. Chemotherapeutics or vaccines to treat or protect against MCF are not available. The disease is characterized by lymphoproliferation and vasculitis, which are considered to be hallmark histological lesions [4]. Besides OvHV-2, MCF can also be caused by several other herpesviruses in the MCF virus (MCFV) group in the genus *Macavirus* [2]. To date ten MCFVs, named for their reservoir species, have been identified and six of these viruses have been associated with disease. They are alcelaphine herpesvirus 1 and 2 (AlHV-1 and -2), caprine herpesvirus 2 and 3 (CpHV-2 and -3), ovine herpesvirus 2 (OvHV-2), and ibex-MCFV [5]. Most mortalities and economic losses from MCF are due to infection with AlHV-1 and OvHV-2 and these two are the most studied MCFVs [2].

Serological assays are usually preferred to screen adult reservoir hosts for their infection status. Due to passive transfer of maternal antibodies in colostrum, serological screening of young animals is not useful until after maternal antibodies wane at approximately three months of age [6]. Current enzyme linked immunosorbent assays (ELISAs), all of which are based on AlHV-1 antigens as the virus can be propagated in culture, can identify MCFV carriers [7–10]. The competitive inhibition ELISA (CI-ELISA) uses a monoclonal antibody, 15A, which recognizes an epitope conserved among all MCFVs and has broad application in detecting MCFV infection [7, 8]. However, none of these serologic assays are OvHV-2 specific. In order to develop a virus-specific assay, a unique target antigen is needed. One such candidate is the previously characterized OvHV-2 glycoprotein Ov8 [11]. Genome sequencing of OvHV-2 revealed that it consists of 74 open reading frames (ORF) [12, 13]. Sixty three ORFs are shared among gammaherpesviruses, nine ORFs are only shared between *Macaviruses* and three are unique to OvHV-2 [12, 13]. In a previous study, one of the nine ORFs, ORF Ov8, was confirmed to be translated from a spliced message into a transmembrane glycoprotein that can enhance cell-cell membrane fusion triggered by OvHV-2 glycoproteins B, H and L [11]. These findings suggest that Ov8 is likely to be a component of the viral envelope and thus expressed during initial lytic virus replication following infection of an animal, making it a potential target for immune responses. ORF Ov8 is localized in the genome between ORFs 50 and 52 [12]. In some gammaherpesviruses, genes localized between ORFs 50 and 52, such as Epstein-Barr virus gp350/220, Kaposi’s sarcoma-associated herpes virus K8.1, and Murid herpesvirus-4 gp150, encode immunogenic glycoproteins that have been used as target antigens for serological assays [14–17]. In this report, we tested the hypothesis that Ov8 is antigenic and induces OvHV-2-specific antibodies. A recombinant Ov8 protein was evaluated for its diagnostic potential to detect OvHV-2-specific antibodies.

## Materials and methods

### Recombinant Ov8 fusion protein

#### Plasmids

Plasmid pJP008 was provided by Li Lin (Laboratory of Cardiovascular Sciences, National Institute on Aging, Baltimore, MD) [18]. Construction of the plasmid pJP007-Ov8 has been described previously [11].

**Fig 1:**
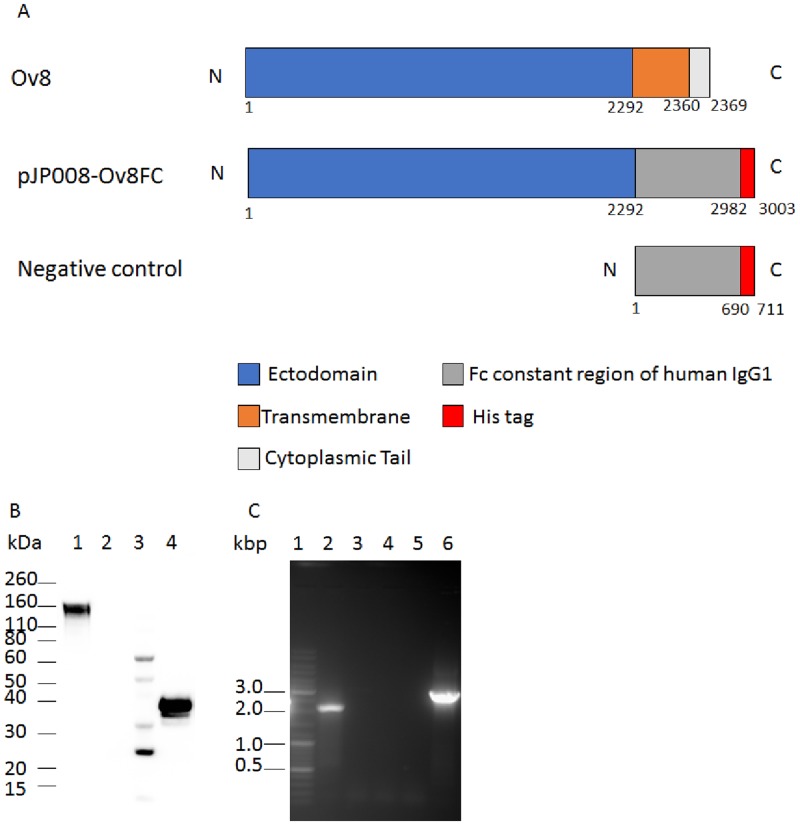
Ov8FC schematic representation and expression. (A) Schematic representation of full length Ov8 (Ov8), truncated Ov8 with Fc region of human IgG1 and His-tag (Ov8FC), and the Fc region of human IgG1 only with His-tag (hIgGFC, Negative control). Numbers below constructs indicate amino acids. (B) Western blot analysis of supernatant from 293-T cells transfected with pJP008-Ov8FC (Lane 1), pJP008 empty plasmid (Lane 2), protein markers (Lane 3) and pJP008-hIgGFC (Lane 4) using mouse monoclonal anti-His antibody. Molecular masses are indicated on the left in kilodalton (kDa). (C) Reverse transcription PCR of cells transfected with pJP008-Ov8FC with and without reverse transcriptase, respectively (Lanes 2 and 3) and pJP008 empty plasmid with and without reverse transcriptase, respectively (Lanes 4 and 5). Control PCR of pJP008-Ov8FC plasmid (Lane 6). DNA marker in kilo base pairs (Lane 1).

A mammalian expression vector containing a truncated Ov8 gene fused to the fragment crystallisable (Fc) region of human IgG1 and 6x His-tag for increased expression and detection of Ov8 in mammalian cells [19], was constructed. Plasmid pUCIDT-AMP-Ov8 fusion protein encoding Ov8 lacking the transmembrane domain and cytoplasmic tail (81538–83829 NC_007646.1) fused with the Fc region of human IgG1 and a 6x His tag at the C-terminus was synthesized by IDT (Integrated DNA Technologies, Inc.) and used as a template for PCR amplification. The PCR reaction consisted of 200 ng plasmid DNA, 1X HotStarTaq master mix (Qiagen), containing HotStarTaq DNA polymerase, buffer and dNTPs, and 0.5 μM of each primer in a total volume of 50 μl. The primers were: sense primer 5’-ACT **GGA TCC** ATG GAT AAC GCT ACC TTA-3’ with a *BamHI* endonuclease restriction site at the 5’ site (bold and underlined) and anti-sense primer 5’- GAG **CTC GAG** TTA ATG GTG GTG GTG ATG AT-3’ with an *XhoI* endonuclease restriction site at the 5’ site (bold and underlined). To amplify the Fc region of human IgG1 only, sense primer 5’-ATT **GGA TCC** GAC AAA ACT CAC ACA TGC-3’ with a *BamHI* endonuclease restriction site at the 5’ site (bold and underlined) and the same anti-sense primer as above were used. The PCR cycle was: 95°C for 5 min, 35 cycles of 95°C for 30 s, 63°C for 30 s, and 72°C for 2.5 min, with a final extension at 72°C for 7 min. Amplified products were detected by agarose gel electrophoresis. Amplicons of 3003 bp and 711 bp, corresponding to the predicted sizes for Ov8 fused to human IgG1 Fc and human IgG1 Fc alone, respectively, were purified (QIAquick gel extraction kit, Qiagen), digested with *BamHI* and *XhoI*, and ligated to the *BamHI* and *XhoI* sites of plasmid pJP008. The resulting plasmids were sequenced to confirm the identity and orientation of the inserts (Eurofins MWG Operon LLC) and designated pJP008-Ov8FC and pJP008-hIgGFC (Fig 1A). Large-scale plasmid preparations were done using the HiSpeed plasmid maxi kit (Qiagen) according to the manufacturer’s protocol.

#### Protein expression

293-T cells obtained from the American Type Culture Collection (ATCC, Manassas, VA), were grown in T-182 flasks with Dulbeco’s Modified Eagle’s Medium (DMEM) with no phenol red (Gibco) supplemented with 10% fetal bovine serum (FBS), 100 U/ml of penicillin, 100 μg/ml of streptomycin, and 1 μg/ml of amphotericin B. At 70% confluency, the medium was changed to 15 ml DMEM with 100 U/ml of penicillin, 100 μg/ml of streptomycin, and 1 μg/ml of amphotericin B and transfection was carried out using polyethylenimine (PEI, Sigma Inc.) at a 1:4 ratio of DNA:PEI (w/v). pJP008-Ov8FC (40 μg) was diluted in 1.5 ml OPTI-MEM (Gibco) and PEI (160 μl) was added to the diluted DNA. The DNA-PEI mix was incubated for 10 min at room temperature then added dropwise to the cells. After 48 hrs, cell culture supernatant was collected, centrifuged at 18000 xg for 10 min at 4°C and concentrated using Centricon Plus-70 with a 100 kDa cut-off (Merck, Millipore Ltd.) according to the manufacturer’s instructions. Plasmid pJP008-hIgGFC was transfected into 293-T cells and concentrated similarly. Concentrated protein was quantified using the Pierce BCA Protein Assay Kit (Thermo Fisher Scientific) according to the manufacturer’s instructions. The concentration of Ov8FC was 3.86 mg/ml and hIgGFC was 3.68 mg/ml.

#### Western blot

Recombinant protein expression was confirmed by Western blot. Concentrated supernatants from cells transfected with pJP008-Ov8FC, pJP008-hIgGFC and empty vector pJP008 were treated with 50 mM Bond-Breaker [tris (2-carboxyethyl) phosphine]at neutral pH (Thermo Fisher Scientific), heated at 95°C for 10 min then separated using SDS-polyacrylamide gel electrophoresis (NuPAGE Novex 4 to 12% Bis-Tris gels, Invitrogen). Separated proteins were transferred to a nitrocellulose membrane using an iBlot 2 gel transfer device (Thermo Fisher Scientific) and the membrane was blocked using phosphate buffered saline (PBS) containing 0.05% Tween 20 (Promega) and 5% non-fat dry milk (Essential Everyday). Membranes were incubated with a horseradish peroxidase (HRP) conjugated anti-His mouse monoclonal antibody (Clontech) diluted 1:7500 in PBS-T (0.05%) with 5% non-fat dry milk for 1 hr to detect His-tagged Ov8FC and hIgGFC. The membrane was washed and processed for detection by chemiluminescence (Clarity Western ECL Substrate, Bio-Rad) and developed on X-ray film.

#### Reverse transcription PCR

Total RNA was purified from 293-T cells transfected with pJP008-Ov8FC using the TRIzol PLUS RNA purification kit (Thermo Fisher Scientific) according to the manufacturer’s instructions. Purified RNA was reverse transcribed to cDNA using the SuperScript IV VILO master mix with ezDNase (Thermo Fisher Scientific) according to the manufacturer’s instructions. PCR was done on the reverse transcribed cDNA using the same PCR conditions and primers described for plasmid pJP008-Ov8FC construction. A no-RT control was used to confirm that there was no DNA contamination. The resulting amplicon was detected by agarose gel electrophoresis, purified and sequenced (Eurofins MWG Operon LLC).

### Anti-Ov8 hyper-immune serum

Four 7-week-old female BALB/c mice (Envigo) were used for testing the antigenicity of OvHV-2 Ov8 and for production of anti-OvHV-2 Ov8 hyper-immune sera. The animals were maintained at Washington State University, Pullman, Washington, in accordance with a protocol (Animal Subjects Approval Form 04380) approved by the Washington State University Institutional Animal Care and Use Committee. Mice were housed as a group with unrestricted access to food and water and were monitored daily by animal care staff.

Anti-Ov8 hyper immune sera were produced by DNA immunization using a gene gun as previously described [20]. Mice were anesthetized via intraperitoneal injection of a solution (0.013 ml/g to 0.02 ml/g) containing xylazine (0.44 mg/ml) and ketamine (6.5 mg/ml). While anesthetized, mice were provided with supplemental warmth and ocular lubricant ointment applied to prevent drying of the eyes. Mice were monitored following anesthesia until they were awake and moving in the cage. Each mouse received a primary and five booster immunizations, at 2–5 week intervals, containing 6 μg of pJP007-Ov8 DNA. Blood from the lateral saphenous vein was collected prior to the primary immunization and 10–11 days after each boost starting from the second boost. Mice were euthanized by CO_2_ asphyxiation followed by exsanguination through cardiac puncture, which was the final sample, and cervical dislocation.

### Plasma and serum samples

The United States Department of Agriculture, Agricultural Research Service, Animal Disease Research Unit in Pullman, Washington, maintains an extensive archive of plasma and serum samples from a variety of species both uninfected and infected with MCFVs. Samples from this archive were selected as defined below to test using the Ov8-specific indirect ELISA.

The MCFV-specific CI-ELISA was used to screen samples from 205 domestic sheep; 106 were positive for anti-MCFV antibodies and 99 were negative. These samples were used in this study.

Eighty-five samples from bison deemed to have MCF, based on the detection of OvHV-2 DNA using real-time PCR [21, 22] and the presence of clinical signs, and 21 samples from uninfected animals, which were healthy and had no detectable OvHV-2 DNA using nested PCR [6, 23], were selected for use in this study.

Sera from rabbits, hartebeests, goats, oryx, and ibex that were infected with AlHV-1, AlHV-2, CpHV-2, Oryx-MCFV and Ibex-MCFV, respectively, as determined by sequencing PCR [24] amplicons and CI-ELISA were also used.

### MCFV CI-ELISA

All of the components used in the CI-ELISA were prepared in-house and the assay performed as previously described [8].

### Ov8 ELISA

Checker-board titration was conducted to optimize the conditions (antigen amount, blocking buffer, primary sera and conjugated secondary antibody dilutions and substrate incubation time) for the assay. Ov8FC and hIgGFC (15.0, 7.5, 3.75, 3.0, 1.875, and 1.5 μg) in ELISA Coating Buffer diluted to 1x (pH 9.3, Biolegend) were used to coat Immulon 4 high bond (HB) 96-well plates (Thermo Fisher Scientific) for 12–18 hrs at 4°C. Supernatant from cells transfected with empty vector pJP008 was diluted 1:10, 1:100, 1:200, and 1:300 in ELISA Coating Buffer diluted to 1x (pH 9.3) and used to coat plates similarly. PBS-T (0.1%) with 20% non-fat dry milk, PBS-T (0.1%) with 3% bovine serum albumin (BSA), or Pierce Protein-Free T20 (TBS) Blocking Buffer (Thermo Fisher Scientific) were used (300 μl/well) to block plates for one hour at room temperature. Predefined positive and negative sheep sera or plasma used as a control in the MCFV CI-ELISA were serially two-fold diluted from 1:5 to 1:160 in blocking buffer. Samples from uninfected bison and bison with MCF that had a high percent inhibition in CI-ELISA were also used in assay optimization. Washes were done five times using 300 μl/well of PBS-T (0.1%) with 0.3 M sodium chloride. Secondary HRP-conjugated antibodies [goat anti-bovine IgG heavy and light chain (H+L)/HRP (Jackson ImmunoResearch Laboratories, Inc.), rabbit anti-sheep IgG (H+L)/HRP (KPL), rabbit anti-goat IgG (H+L)/HRP (KPL), goat anti-rabbit IgG (H+L)/HRP (Jackson ImmunoResearch Laboratories, Inc.), and donkey anti-mouse IgG/ HRP (Abcam)] were titrated using the manufacturers’ recommendations as a starting point. TMB Microwell Peroxidase Substrate (SurModics) was then added at 100 μl/well and incubated for 10 mins and the reaction was stopped by addition of 100 μl/well of 0.18 M sulfuric acid. The plate was then read at 450 nm optical density using an ELISA plate reader (Multiscan MCC 350, Thermo Scientific). After assay optimization, all samples were tested in duplicate one time.

### Statistical analysis

The receiver operating characteristic (ROC) plot was generated using SigmaPlot version 11 (Systat Software, Inc.). Kappa values were calculated using GraphPad QuickCalcs (https://www.graphpad.com/quickcalcs/kappa1.cfm). Descriptive statistics were calculated using SigmaPlot version 11 (Systat Software, Inc.).

## Results

### Ov8FC expression

Western blot analysis was done on supernatant of cells transfected with plasmid pJP008-Ov8FC and pJP008-hIgGFC using mouse monoclonal anti-His antibody to confirm recombinant protein expression. Bands at 160 kDa representing glycosylated Ov8FC (Fig 1B, lane 1) and at 45 kDa representing hIgGFC (Fig 1B, lane 4) were detected, while no bands were detected in supernatants from cells transfected with pJP008 alone (Fig 1B, lane 2). The Ov8FC and hIgGFC bands confirmed that the proteins were secreted in the supernatant.

Because Ov8 protein is known to be translated from a spliced mRNA [11], RT-PCR was done to ensure that the removal of the transmembrane domain and the cytoplasmic tail coding regions didn’t affect the mRNA splicing. PCR on the cDNA from cells transfected with pJP008-Ov8FC and agarose gel electrophoresis of the amplicon revealed a band of 2055 bp which corresponds to the Ov8FC gene with no intron showing that the mRNA was indeed spliced (Fig 1C). Sequencing of the amplicon showed splicing at the previously predicted sites (82756 and 83704 NC_007646.1) [11, 12].

### Ov8 protein antigenicity

**Fig 2:**
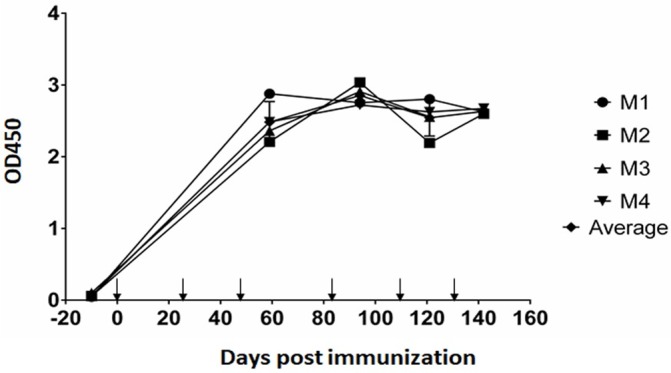
Ov8 antigenicity in mice after DNA immunization. Serum samples from four mice that were DNA immunized with pJP007-Ov8 were tested in Ov8 ELISA. The samples were collected at 59, 94, 121, and 142 DPI. M1, 2, 3, and 4 refer to each of the 4 mice. Arrows show the days the mice were immunized (prime and 5 boosts at days 27, 49, 83, 111, and 135). Bars indicate the standard deviation of the mean titers at each time point.

Sera collected from four mice immunized with plasmid pJP007-Ov8 and diluted 1:100 were tested in the optimized Ov8 ELISA. Pre-immune sera were used as negative controls [OD_450_ mean 0.087, standard deviation (SD) 0.0025] and the cut-off point was determined as the mean OD + 3 SD (0.094). All four mice developed an Ov8 antibody response following DNA immunization (Fig 2 and S1 Table). The first serum sample collected at 59 days post-immunization (DPI) was positive for all mice. Subsequent samples, obtained at 94, 121, and 142 DPI, were also positive and at consistent levels (Fig 2). To check for cross-reactivity with AlHV-1 the sera from the mice immunized with Ov8 were tested using the Ov8 ELISA protocol in AlHV-1 antigen coated ELISA plates. The pre- and post- immune sera had similar OD values (0.147 and 0.177).

### Ov8 ELISA optimization

Testing of parameters to maximize the signal to noise ratio of the Ov8 ELISA resulted in the following protocol. Immulon 4 HB 96-well plates were coated with 3 μg of protein (50 μl/well) in 1x ELISA Coating Buffer at 4°C for 12–18 hrs. Excess protein was removed and plates were blocked by the addition of TBS (300 μl/well) for 1 hr on a rotating platform (500 rpm). The blocking buffer was removed and the plates dried at room temperature for 2 hrs. Sealed dried plates were stored at 4°C with desiccant and used after 24 hrs up to one week later. Sheep sera diluted 1:100, bison sera diluted 1:20, and the other sera diluted 1:40 in blocking buffer (50 μl/well) were added to the coated plate and incubated at room temperature for 1 hr. Sera were removed and the plate rinsed five times with 300 μl/well wash buffer [PBS-T (0.1%), 0.3M NaCl]. HRP-conjugated secondary antibodies diluted in blocking buffer [anti-sheep IgG (H+L) and anti-bovine IgG (H+L) diluted 1:20,000, and anti-rabbit and anti-mouse diluted 1:4000] were added (50 μl/well) and incubated for 1 hr at room temperature. Conjugated antibodies were removed and the plate rinsed as for primary sera. TMB Microwell Peroxidase Substrate was added (100 μl/well). After 10 min incubation at room temperature, the reaction was stopped by addition of 0.18 M sulfuric acid (100 μl/well). The plate was then read at 450 nm optical density using an ELISA plate reader. Interplate coefficients of variation (CV) of positive control sheep plasma, from the MCFV CI-ELISA, of eight plates had a median value of 0.67%, maximum and minimum values of 0.77% and 0.61%, respectively, and a range of 0.16%. These results indicate that the Ov8 protein is stable for at least one week after coating of microtiter plates.

### OvHV-2 specificity of Ov8 ELISA

Serum samples from animals infected with Ibex-MCFV, CpHV-2, AlHV-1, AlHV-2, Oryx-MCFV or OvHV-2, as previously confirmed by MCFV CI-ELISA and PCR, were examined using the Ov8 ELISA. All samples from uninfected animals and animals infected with MCFVs other than OvHV-2 were negative in the Ov8 ELISA, thus showing a 100% negative predictive value (Table 1, S1 Fig). Intraplate CVs of samples from animals infected with MCFVs are shown in S2 Fig. They ranged from 0.83–6.46% for OvHV-2-infected sheep samples to 7.38–28.56% for samples from AlHV-1-infected rabbit samples.

**Table 1:** Ov8 ELISA specificity using reference serum samples pre-defined as positive or negative by CI-ELISA and PCR.

	Ov8 ELISA	
Animal species ^a^	^b^ Anti-MCFV antibody positive sera	^c^ Anti-MCFV antibody negative sera
**Ibex-Nubian**	0/8 (Ibex MCFV)	8/8
**Goat**	0/3 (CpHV-2)	3/3
**Rabbit**	0/7 (AlHV-1)	7/7
**Hartebeest**	0/3 (AlHV-2)	n/a^d^
**Oryx**	0/8 (Oryx-MCFV)	8/8
**Sheep**	10/10 (OvHV-2)	10/10

^a^ Animal species the serum samples were taken from.

^b^ Number of serum samples that were positive in Ov8 ELISA from a total of CI-ELISA positive samples tested. The MCFV detected in the infected animal species tested is indicated in parenthesis.

^c^ Number of serum samples that were negative in Ov8 ELISA from a total of CI-ELISA negative samples tested.

^d^ No CI-ELISA negative samples available.

### Ov8 ELISA performance

#### Ov8 ELISA comparison with CI-ELISA on sheep sera

Two hundred and five sheep serum samples predefined by CI-ELISA as positive (106 sera) or negative (99 sera), were tested in Ov8 ELISA. From all samples tested, 106/106 were positive in Ov8 ELISA and 98/99 were negative in Ov8 ELISA (Table 2, S3 Fig). The assay showed 100% diagnostic sensitivity, 98.99% diagnostic specificity, 99.07% positive predictive value, and 100% negative predictive value relative to the CI-ELISA. It also showed a very high agreement with the CI-ELISA (kappa = 0.990 and 95% CI = 0.971–1.000). A receiver operating characteristic (ROC) plot of the data gave an area = 1.00 (S4 Fig).

**Table 2:** Results for sheep sera as tested by the MCFV CI-ELISA and the Ov8 ELISA.

	CI-ELISA positive	CI-ELISA negative	Total
**Ov8 ELISA positive**	106	1	107
**Ov8 ELISA negative**	0	98	98
**Total**	106	99	205

#### Ov8 ELISA comparison with CI-ELISA on bison sera

One hundred and six serum samples from bison that either showed MCF clinical signs and had positive OvHV-2 PCR results (85 positive samples) or showed no clinical signs and had negative PCR results (21 negative samples) were tested side by side using CI-ELISA and Ov8 ELISA. Ov8 ELISA detected 48/85 samples as positive while the CI-ELISA detected 49/85 samples as positive (Table 3, S5 and S6 Figs). Both assays detected 21/21 samples as negative (S5 and S6 Figs). Considering only the positive samples, Ov8 ELISA had 56.7% diagnostic sensitivity, 100% positive predictive value, and 0% negative predictive value relative to PCR, the gold standard for diagnosing MCF in clinically susceptible animals. Similarly, the CI-ELISA had 57.6% diagnostic sensitivity, 100% positive predictive value, and 0% negative predictive value. Comparison of the two ELISA assays with each other showed fair agreement (kappa = 0.400, 95%CI = 0.203–0.596).

**Table 3:** Antibody detection by Ov8 ELISA and MCFV CI-ELISA in 85 serum samples from bison with clinical MCF confirmed by PCR.

	CI-ELISA positive	CI-ELISA negative	Total
**Ov8 ELISA positive**	36	12	48
**Ov8 ELISA negative**	13	24	37
**Total**	49	36	85

## Discussion

Current serological assays to screen potential OvHV-2 carriers rely on cross-reactivity of anti-OvHV-2 antibodies with AlHV-1. In order to develop an OvHV-2-specific assay, a unique viral target antigen is needed. A recombinant protein containing part of Ov8, a unique OvHV-2 protein, was selected to test as an antigen target in an indirect ELISA format. A previous study characterized the novel OvHV-2 glycoprotein Ov8 [11]. Ov8 was shown to be highly glycosylated and was able to enhance cell-cell membrane fusion mediated by conserved viral core fusion proteins [11]. We hypothesized that Ov8 is antigenic and anti-Ov8 antibodies are specific for OvHV-2, thus making it a possible antigen for a serological assay specific for OvHV-2. To test the antigenicity of Ov8, mice were immunized with plasmid DNA encoding a full length Ov8. All four mice developed antibodies against Ov8 thus demonstrating the antigenicity of the protein. To further evaluate the uniqueness of Ov8 for OvHV-2, the predicted amino acid sequence of Ov8 was compared with A8 of AlHV-1 and AlHV-2, the only complete annotated MCFV genomes available in addition to OvHV-2 [12, 13, 25, 26], revealing identities of 49% and 50%, respectively. Cross-reactivity of the mouse anti-Ov8 serum with AlHV-1 was tested by indirect ELISA using 96-well plates coated with AlHV-1 antigens. Pre- and post-immune sera produced similar OD values showing that the mice anti-Ov8 polyclonal sera had no cross-reactivity to AlHV-1.

The Ov8 ELISA was developed using a recombinant glycoprotein Ov8 as the target antigen. None of the sera from animals infected with MCFVs other than OvHV-2 showed reactivity to Ov8 in the ELISA. These results further indicate Ov8 is an OvHV-2-specific antigen and can be used for a serological assay to detect OvHV-2-specific antibodies.

Sheep are the reservoir species for OvHV-2 and remain infected for life. Anti-viral antibodies are reliably detected in OvHV-2 infected sheep using the MCFV CI-ELISA [7, 8, 27]. A panel of sera from uninfected and infected sheep, as defined by the CI-ELISA, was used to test the performance of the Ov8 ELISA. The results demonstrated that the Ov8 ELISA can be used to specifically detect OvHV-2 infection in sheep. In the future, a validated Ov8 indirect ELISA could be useful to mixed species operations like zoos and game farms by allowing screening of potential OvHV-2-carrier animals and establishment of appropriate measures to prevent virus transmission to clinically susceptible species.

The gold standard for diagnosing MCF in clinically susceptible species, such as bison, is the presence of histological lesions consistent with the disease and PCR. Serology has not been useful for diagnosing MCF. Previous studies documented some bison with clinical signs of MCF had detectable OvHV-2 DNA levels using PCR but no anti-MCFV antibodies detected by CI-ELISA [28]. Bison are known to show weak immune responses when infected with OvHV-2 [29], which might explain why some bison don’t develop antibodies detectable by CI-ELISA. However, because the CI-ELISA uses competition against a monoclonal antibody to a single epitope, the assay could be missing certain serological responses to OvHV-2. Therefore, we tested serum samples from bison that had clinical signs of MCF and were positive for OvHV-2 DNA in tissues, in both the CI-ELISA and Ov8 ELISA. False negative results were obtained in both assays, but the Ov8 ELISA detected antibodies in 33.3% (21/36) of samples that were scored negative using CI-ELISA. This shows that at least some animals that didn’t develop antibodies against the epitope detected by mAb 15A in CI-ELISA developed antibodies against Ov8, providing a potential target antigen for future vaccine development.

This study was undertaken to determine whether the OvHV-2 glycoprotein Ov8 was a suitable candidate to be used in a serological assay. The results of the study confirmed that Ov8 is a reasonable target for such an assay. Future work will focus on the validation of the indirect Ov8 ELISA for research and diagnostic purposes.

## Supporting information

S1 Fig Ov8 ELISA does not cross react with antibodies against other MCFVs. Corrected optical density values of Ov8 ELISA on samples from animals uninfected (A) or infected (B) with malignant catarrhal fever viruses as predetermined by PCR and CI-ELISA. Solid horizontal line indicates cut off value. I = Ibex, O = Oryx, H = Hartebeest, G = Goat, R = rabbit, S = domestic sheep.
(TIF)

S2 Fig Intraplate coefficients of variance (CV). Percent CV of samples from animals infected with MCFVs in the Ov8 ELISA.
(TIF)

S3 Fig Ov8 ELISA sheep samples. Ov8 ELISA on OvHV-2 uninfected and infected sheep expressed as percent positive control. Solid horizontal line indicates cut off value.
(TIF)

S4 Fig ROC plot Ov8 ELISA sheep samples. Comparison of Ov8 ELISA with CI-ELISA. A = area under the curve.
(TIF)

S5 Fig Ov8 ELISA bison samples. Corrected optical density values of Ov8 ELISA on samples from uninfected bison (top graph) and bison with malignant catarrhal fever (bottom graph). Solid horizontal lines indicate cut off values.
(TIF)

S6 Fig CI-ELISA bison samples. Percent inhibition in CI-ELISA of samples from uninfected bison (top graph) and bison with malignant catarrhal fever (bottom graph). Solid horizontal lines indicate cut off values.
(TIF)

S1 Table Ov8 ELISA mice sera. OD_450_ values days post immunization (DPI). Values are averages of duplicate measurements.
(XLSX)
