# Supplementary figures and images for: Evaluation of glycoprotein Ov8 as a potential antigen for an OvHV-2-specific diagnostic assay
Source: PLoS One. 2018 Jul 2;13(7):e0200130. doi: 10.1371/journal.pone.0200130 (PMC6028116; doi:10.1371/journal.pone.0200130)

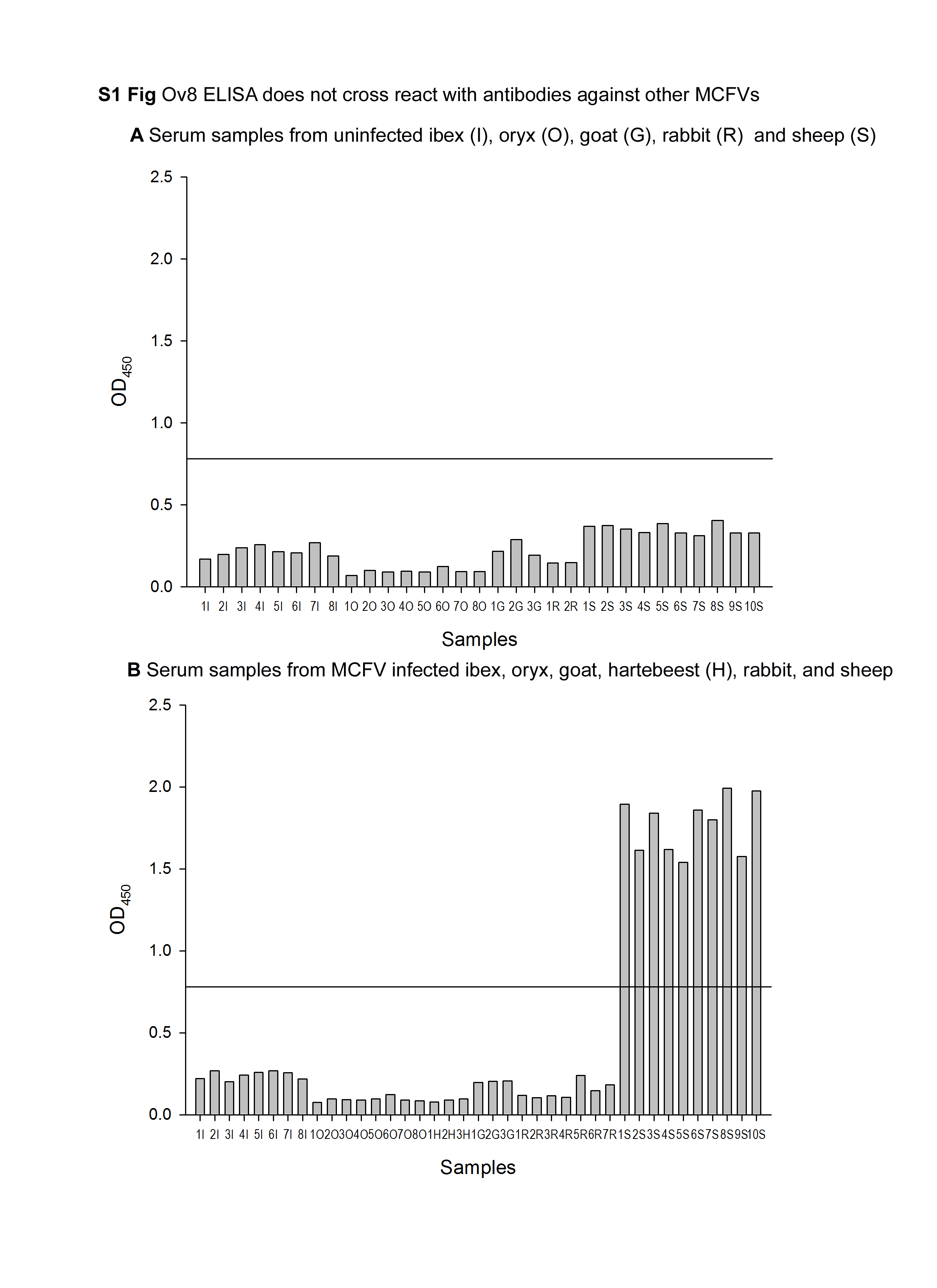

Supplement: S1 Fig — Corrected optical density values of Ov8 ELISA on samples from animals uninfected (A) or infected (B) with malignant catarrhal fever viruses as predetermined by PCR and CI-ELISA. Solid horizontal line indicates cut off value. I = Ibex, O = Oryx, H = Hartebeest, G = Goat, R = rabbit, S = domestic sheep. (TIF) [file pone.0200130.s001.tif]

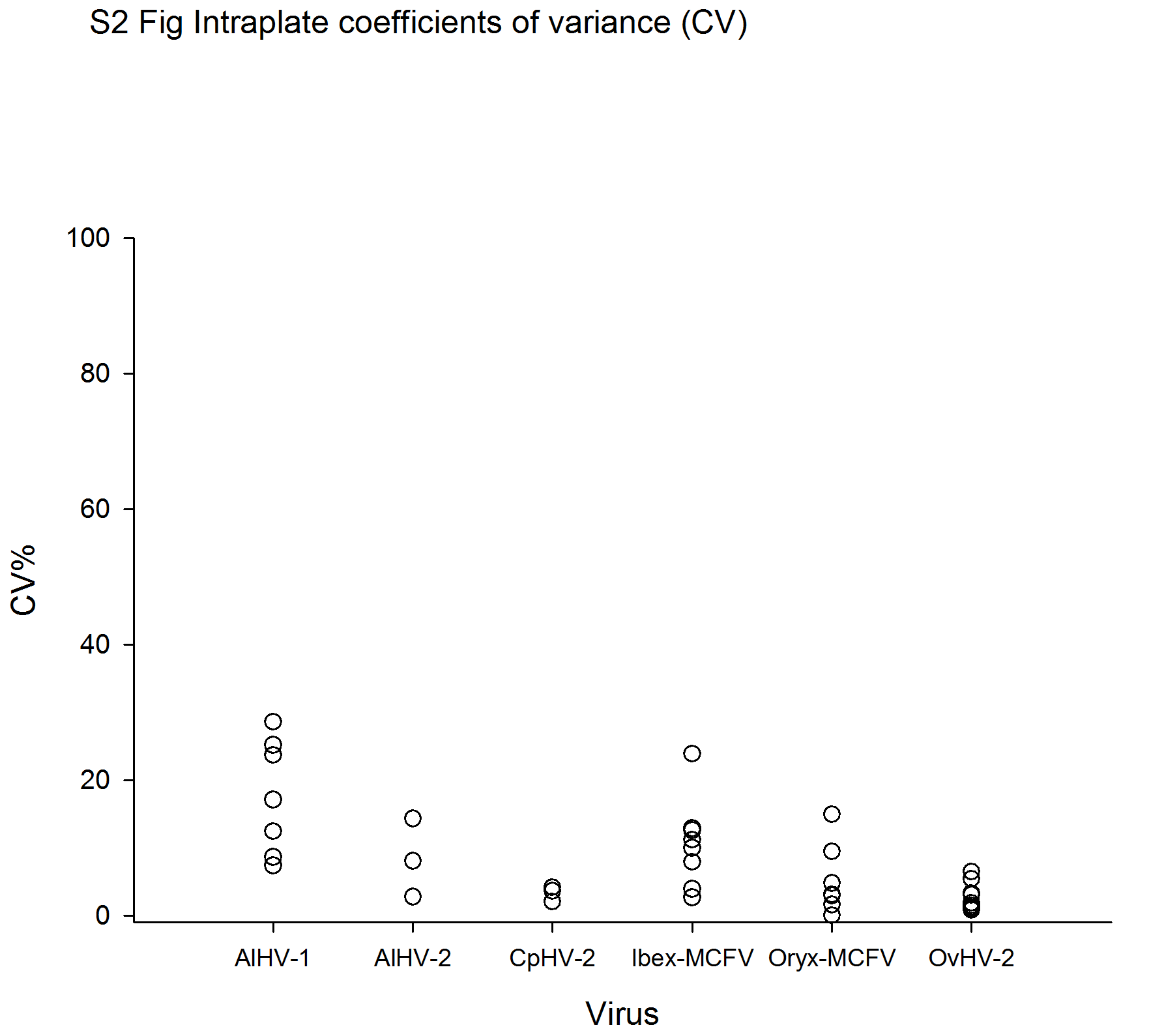

Supplement: S2 Fig — Percent CV of samples from animals infected with MCFVs in the Ov8 ELISA. (TIF) [file pone.0200130.s002.tif]

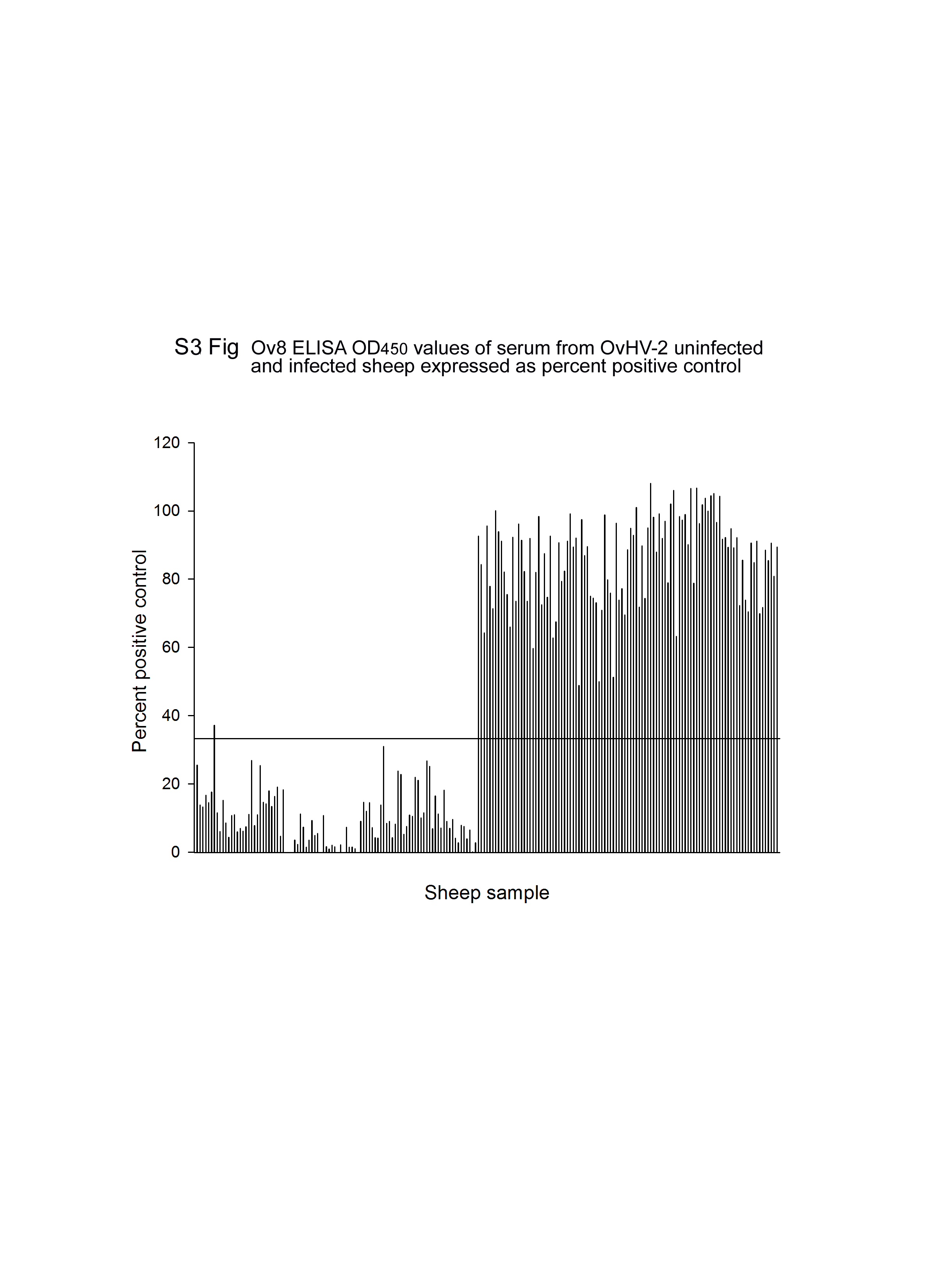

Supplement: S3 Fig — Ov8 ELISA on OvHV-2 uninfected and infected sheep expressed as percent positive control. Solid horizontal line indicates cut off value. (TIF) [file pone.0200130.s003.tif]

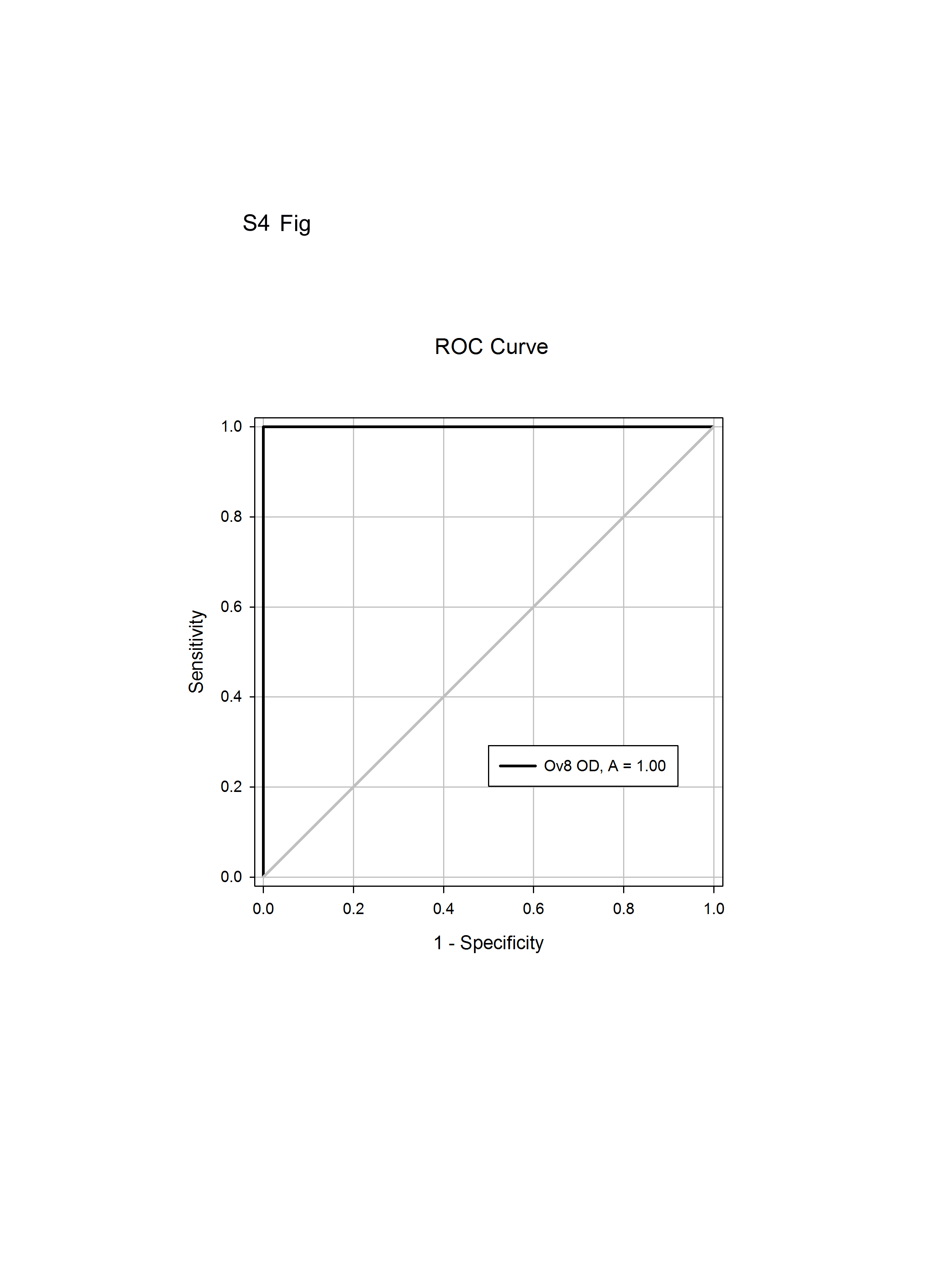

Supplement: S4 Fig — Comparison of Ov8 ELISA with CI-ELISA. A = area under the curve. (TIF) [file pone.0200130.s004.tif]

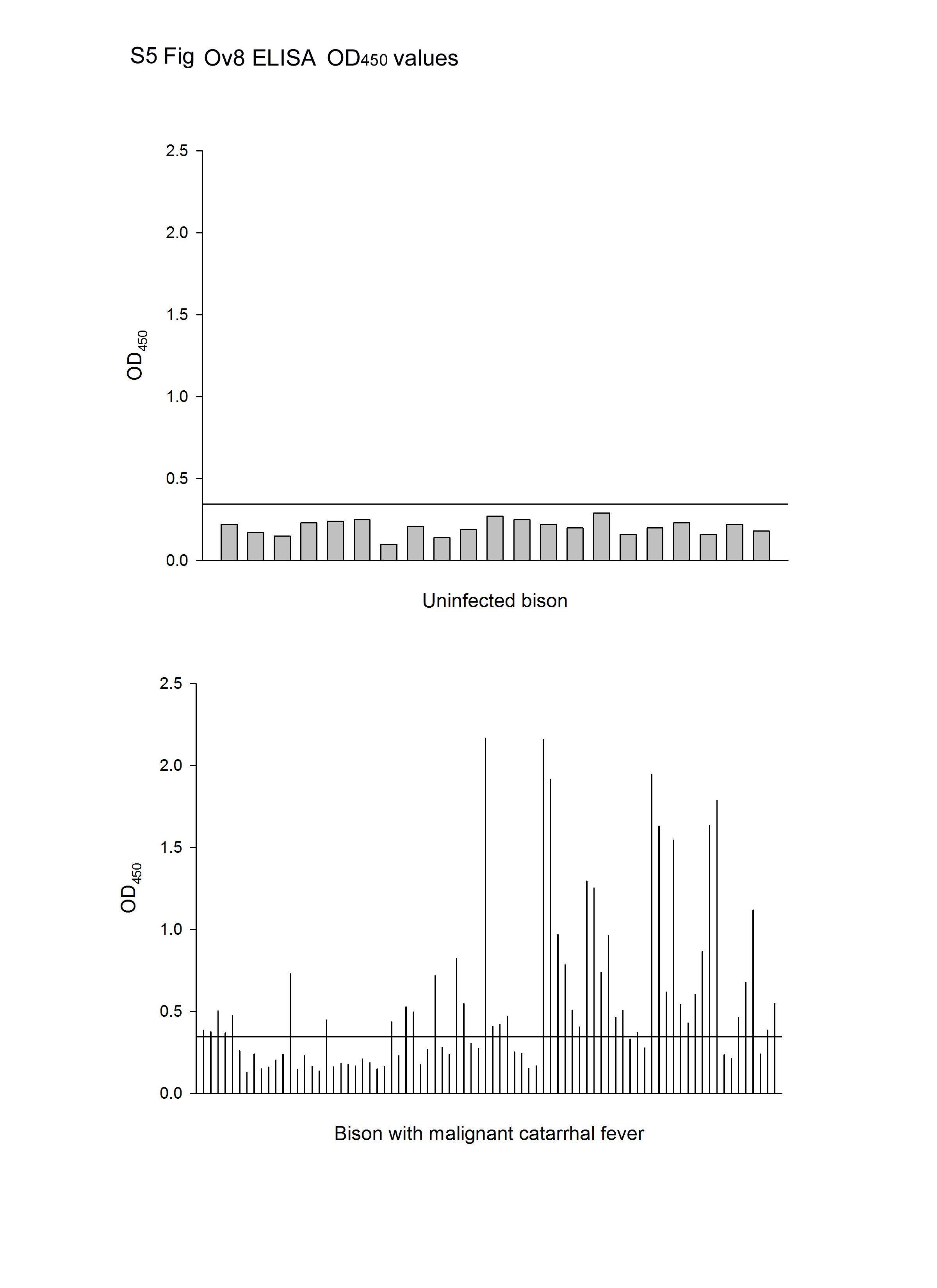

Supplement: S5 Fig — Corrected optical density values of Ov8 ELISA on samples from uninfected bison (top graph) and bison with malignant catarrhal fever (bottom graph). Solid horizontal lines indicate cut off values. (TIF) [file pone.0200130.s005.tif]

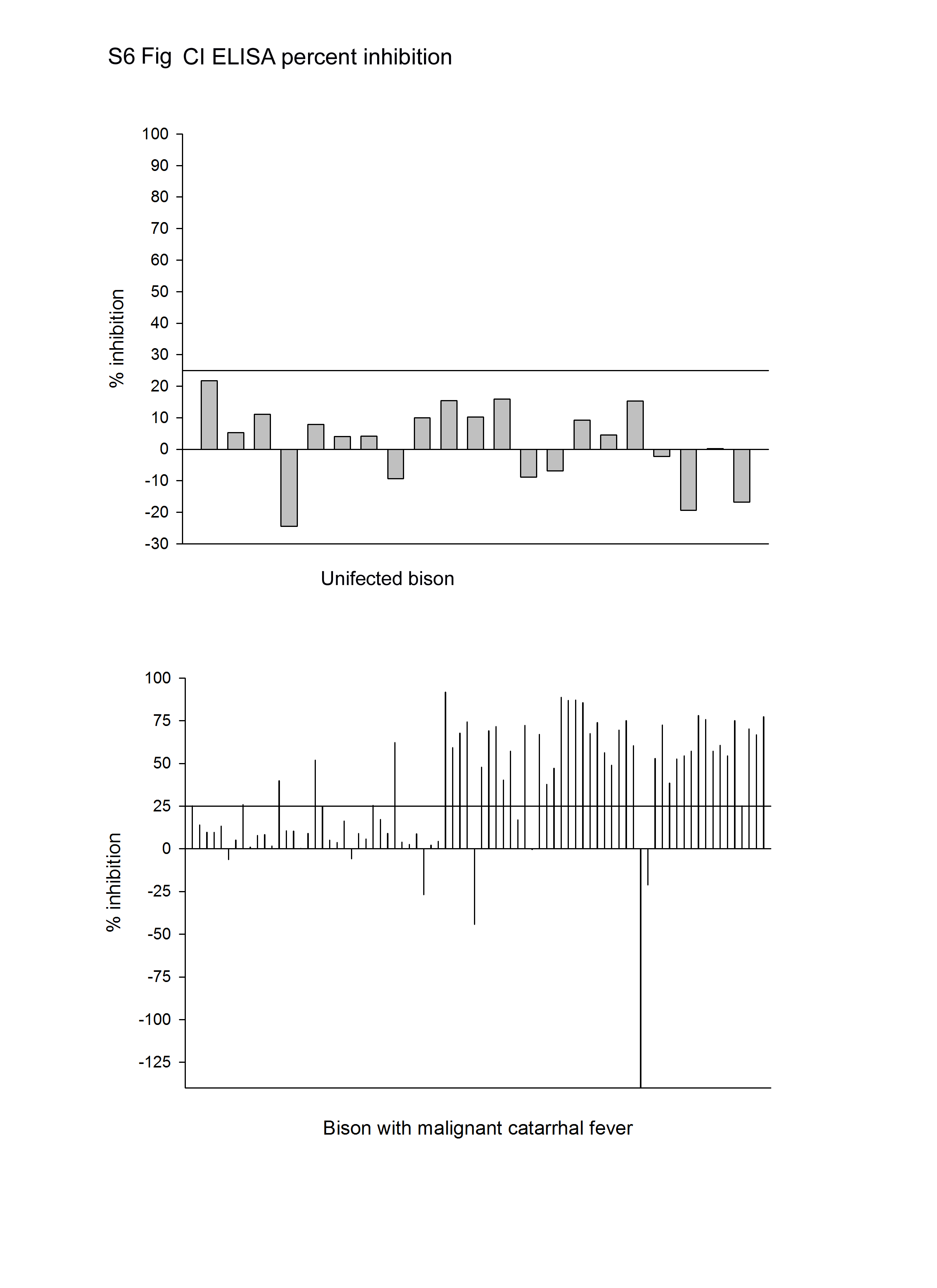

Supplement: S6 Fig — Percent inhibition in CI-ELISA of samples from uninfected bison (top graph) and bison with malignant catarrhal fever (bottom graph). Solid horizontal lines indicate cut off values. (TIF) [file pone.0200130.s006.tif]
